# Unveiling the Link Between Inflammation and Adaptive Immunity in Breast Cancer

**DOI:** 10.3389/fimmu.2019.00056

**Published:** 2019-01-29

**Authors:** Tadeo Enrique Velazquez-Caldelas, Sergio Antonio Alcalá-Corona, Jesús Espinal-Enríquez, Enrique Hernandez-Lemus

**Affiliations:** ^1^Computational Genomics Division, National Institute of Genomic Medicine, Mexico City, Mexico; ^2^Centro de Ciencias de la Complejidad, Universidad Nacional Autónoma de México, Mexico City, Mexico; ^3^Department of Ecology and Evolution, Erman Biology Center, The University of Chicago, Chicago, IL, United States

**Keywords:** inflammation, breast cancer, adaptive immunity, network biology, systems biology

## Abstract

Inflammation has been recognized as an important driver in the development and growth of malignancies. Inflammatory signaling in cancer emerges from the combinatorial interaction of several deregulated pathways. Pathway deregulation is often driven by changes in the underlying gene regulatory networks. Confronted with such complex scenario, it can be argued that a closer analysis of the structure of such regulatory networks will shed some light on how gene deregulation led to sustained inflammation in cancer. Here, we inferred an inflammation-associated gene regulatory network from 641 breast cancer and 78 healthy samples. A modular structure analysis of the regulatory network was carried out, revealing a hierarchical modular structure. Modules show significant overrepresentation score *p*-values for biological processes unveiling a definite association between inflammatory processes and adaptive immunity. Other modules are enriched for T-cell activation, differentiation of CD8^+^ lymphocytes and immune cell migration, thus reinforcing the aforementioned association. These analyses suggest that in breast cancer tumors, the balance between antitumor response and immune tolerance involving CD8^+^ T cells is tipped in favor of the tumor. One possible mechanism is the induction of tolerance and anergization of these cells by persistent antigen exposure.

## 1. Introduction

Breast cancer is the most frequently diagnosed malignancy in women worldwide (1). Given its prevalence, a great effort has been made to understand the mechanisms that lead to its development including genetic analysis, mutation status for known oncogenes ad tumor suppresors, expression status of known associated receptors like ER and ERBB2 (HER2), as well as transcriptomic assays as exemplified in Cancer Genome Atlas Network (2). Breast cancer tumors appear to be highly heterogeneous in all these aspects, nevertheless a shared set of characteristics are recognizable during tumor development, which are widely known as the hallmarks of cancer (3, 4). In this view, cancerous phenotypes result from a complex of interacting biological processes or pathways that are subverted in favor of tumor survival, growth and invasion.

Inflammation is necessary to maintain homeostasis. Nontheless, alterations in the course of inflammatory response can lead to pathological states, specially when it cannot be resolved and becomes chronic. The effects of inflammation include changes in tissue properties like blood vessel permeability, alteration of extra cellular matrix and the initiation of immune responses that involve recruitment, proliferation and differentiation of innate as well as adaptive immune cells, actions that are coordinated in a combinatorial way by cytokine and chemokine production (5, 6). There is a strikingly similar scenario when we look at tumors.

The role of inflammation in tumor development is far from simple. It is known that acute inflammatory response is an important component in the spontaneous elimination of tumors (7). Also there are examples in which correlation exists between chronic inflammation and some types of cancer like colon, gastric, esophageal, thyroid and breast cancers (8–12). Although it is recognized that chronic inflammation has a role in the tumorigenic process, it is not well understood how or why inflammation is also necessary for tumor maintenance. In particular, breast cancer tumors show significant infiltrates of immune system cells (13). Infiltrates can vary in the amount and specific types of immune cells they contain. Some evidence points to a relation between mixtures of immune cells in breast cancer subtypes and patient outcome in relation to metastasis recurrence and survival time (5, 14). This suggests that inflammatory response in conjunction with the immune system plays an important role on breast cancer maintenance.

Considering the intricate set of relationships that inflammation and immunity play in a complex phenotype such as a breast cancer, an integrative approach capable of capturing at least part of the complexity of biological processes related to inflammation results appealing. In this context, the construction of gene regulatory networks via the association between gene expression levels, offers us a valuable view on how groups of genes are being collectively coordinated.

The modeling of the gene regulatory structure through the use of complex networks (15–17) opens up the use of techniques to interrogate network structure (15), as is the case with network modularity (18, 19). Resulting modules can then be associated biological functions (20). To find the modular structure in a network is a non-closed problem, which has been subject of intense research in network theory (15, 21). Furthermore, the applications of finding modular structure in real-world networks has also been matter of interest in several fields, including social (22) and biological systems (20, 23).

Previous work from our group has shown how network modularity structure is associated to biological functionality in transcription factor networks (20) and distinctive network structure for each of the major breast cancer molecular subtypes (24). Furthermore, by means of an automated analysis of network inference, module detection and enrichment analysis we have been capable to detect specific processes in breast cancer molecular subtypes.

In this work, we approach the entangled nature of the inflammatory process in the maintenance of breast cancer phenotype through the inference of an inflammation-related gene regulatory network and their associated genes (their immediate or first neighbors in network terminology), then finding the modular structure of the network and associated biological processes for each module.

We found a hierarchical modular structure in transcriptional networks associated to inflammatory response, where genes tend to be connected to others with similar differential expression patterns: overexpressed genes are more connected between them, as well as underexpressed ones. Modules of the network are mainly associated to immune system, extracellular matrix and cell adhession.

A comprehensive integrative framework allows us to observe a broader landscape of how inflammation may have influence in the establishment of pathological phenotypes. At the same time, this exploratory approach could help to direct research toward more specific questions about therapeutic options.

## 2. Materials and Methods

### 2.1. Data Acquisition

**Figure 1:**
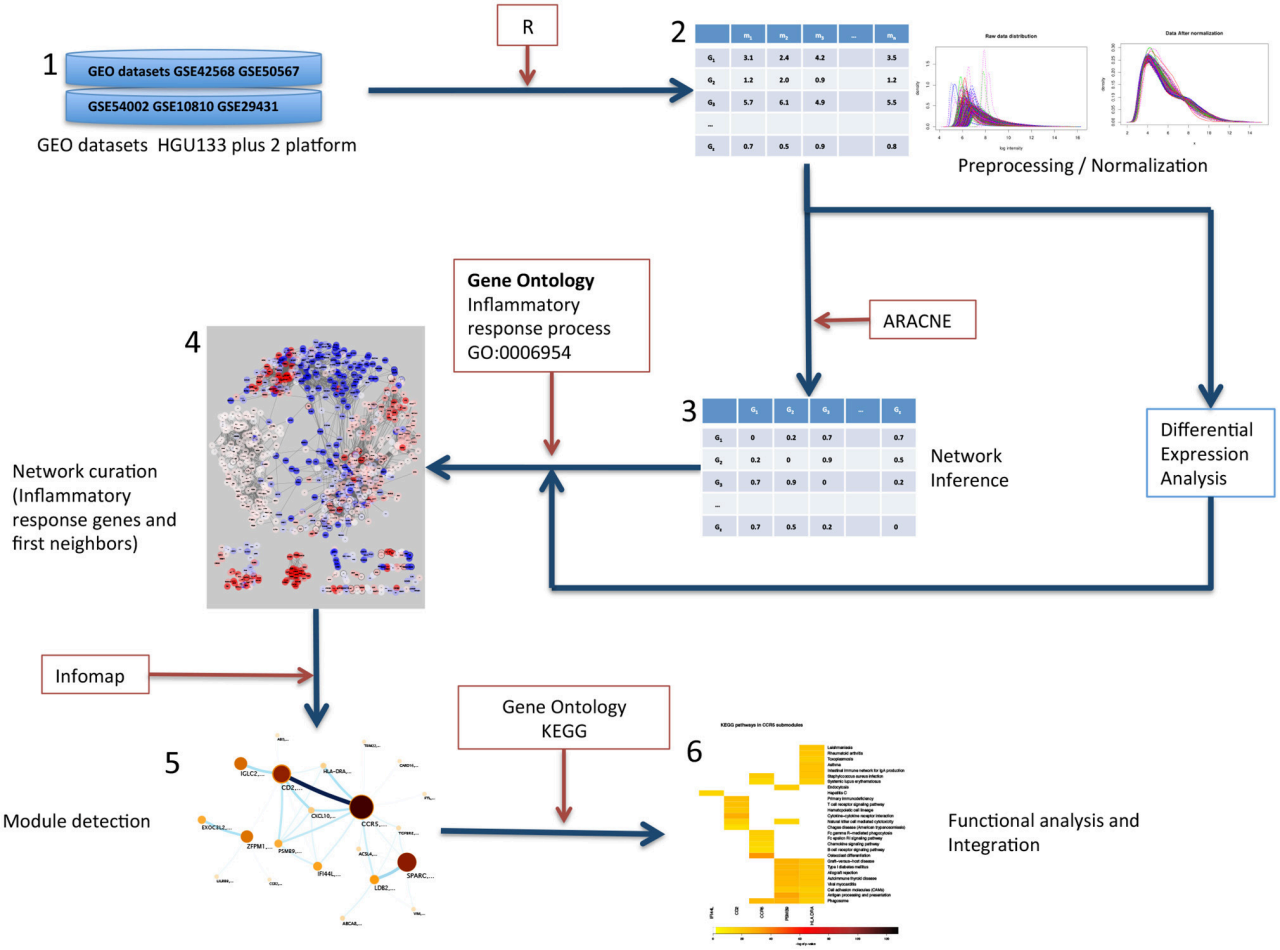
Graphical description of the methods followed in this work. (1) Gene expression datasets from GEO were selected. All datasets contained normal tissue samples and primary, untreated tumor samples. (2) Datasets were preprocessed, merged and normalized to obtain an expression matrix (Rows correspond to genes and columns correspond to samples). (3) Tumor samples expression matrix was used to calculate all statistical dependencies with the MI function between pairs of genes to construct the network. (4) The network was filtered to obtain the top 10,000 interactions ranked by MI value of genes in the inflammatory response process and first neighbors. (5) The inflammation network was analyzed to detect modules via the Infomap algorithm. (6) The resulting modules were tested for functional enrichment of Gene Ontology.

A graphical description of the followed pipeline is depicted in Figure 1. We used a curated database that consists of 719 microarray assays of the platform Affymetrix HGU 133 plus 2 from 641 untreated primary breast cancer samples and 78 healthy mammary tissue samples. The data was obtained from five separate data series deposited in the Gene Expression Omnibus https://www.ncbi.nlm.nih.gov/geo/(25), each data series contais case and control samples (Table 1). Raw data. CEL files were pre-processed and normalized as described in https://github.com/CSB-IG/rnw/tree/master/normalization-preprocessing. The resulting expression matrix was used as input to perform network inference. Differential expression (DE) analysis contrasting tumors to healthy mammary tissue was performed with the normalized expression matrix using Empirical Bayes statistic from Bioconductor's library Limma (30) for the R statistical software.

**Table 1:** GEO identifier and references for the data used here.

**GEO ID Series**	**Tumors**	**Controls**	**Description**	**References**
GSE42568	104	17	Gene expression profiling of 104 breast cancer and 17 normal breast biopsies.	(26)
GSE50567	35	6	Flash-frozen surgical samples obtained during mastectomy from patients without neoadjuvant chemotherapy	(27)
GSE54002	417	16	Mammary gland cells were captured from clinical tissues of breast cancer patient by Laser Capture Microdissection	(28)
GSE10810	31	27	Samples were taken from patients before their treatment.	(29)
GSE29431	54	12	Primary breast carcinomas to identify a gene expression profile for breast tumors based on HER2 status.	Not published
Total	641	78		

*The first column indicates the GEO accession number, the second and third indicate the number of cases/controls. The fourth column is a short description of the samples and the fifth is the asociated reference*.

### 2.2. Network Inference

Since we are interested in the integration of inflammatory response with other biological processes in the breast cancer phenotype, we used a gene regulatory network approach to obtain groups of associated genes. Although numerous algorithms and correlation measures can be used to infer networks based on expression data (31), it can be formally demonstrated that for datasets with more variables than samples and inherently noisy data, the best statistical dependency measure is Shannon's mutual information (MI) since it is capable to detect non-linear dependencies and is not affected by data transformations like normalization (it is reparameterization invariant) (32–34). A network in this context is a mathematical object composed of a colection of nodes that represent genes, and a colection of edges that represent the statistical dependency between pairs of genes.

We used MI calculations implemented in the ARACNe algorithm (34) to calculate pairwise statistical dependency for all gene pairs in the platform (MI threshold set with *p* ≤ 1) which gives us a completely connected graph (a network where all possible interactions exist) for the whole-genome. Our network contains only the top 10,000 most stringent interactions between genes from the inflammatory response and their first neighbors, which correspond to 0.005% of possible correlated gene pairs. The reasoning behind this is that the genes defining the phenotype must have the strongest statistical dependencies between them, hence are at the top of the ordered list, and also in order to minimize the effect of false positives. Since we are interested in inflammation, to start with a list of genes of interest can help us to discover other genes associated to them at the transcriptional level. This however does not guarantee that we recover all genes in the list but the ones within our imposed threshold.

The network was curated starting from the complete graph using a reference gene list of the inflammatory process obtained from Gene Ontology (Inflammatory response process GO:0006954) with the following procedure: First, we ranked all interactions in descending order (largest to smallest MI value). Second, we searched for the genes sharing the strongest interactions with those in the inflammatory response process. This was done by filtering those interactions where at least one gene is part of the inflammatory response. From these interactions we took the top 10,000 interactions and obtained the names of all the genes involved which comprises the list of inflammatory response and associated genes. Third, because we wanted to recover interactions between inflammation gene first neighbors, we extracted from the network the top 10,000 interactions between the genes of the list (inflammation and first neighbors).

### 2.3. Hierarchical Module Detection

Gene Regulatory Networks, such as those we inferred, contain associations in the scale of hundreds of genes connected by thousands of interactions. Nevertheless, the connectivity patterns are not uniform through the structure of the network. Highly interconnected groups of genes may be indicative of coordinated expression associated with biological functions, making relevant the identification of such groups. This approach has been previously proved by our group in the context of GRNs on breast cancer subtypes (23). We showed that gene modules are representative of distinct and meaningful biological functions.

Thus, in order to find to find connectivity patterns in our network, here we used Infomap (19), a well-known flow-based information clustering method to determine the modular structure of complex networks. Likewise, we use the expanded version of Infomap to find a finer modular structure over the two-level modules using the hierarchical version of the map equation (35).

Using the hierarchical map equation it possible to exploit the fact that the modules in a network are themselves organized into submodules and sub-submodules which can reveal a richer multilevel organization. This approach has been successfully applied as well in the case of GNRs associated with Her2+ cancer subtype (36).

### 2.4. Functional Analysis

The number of genes contained in each module can amount to several hundred. Additionally, we have to deal with the fact that individual genes can be annotated for more than one function or pathway. To obtain biological insights from gene sets like these, we use statistical over representation analysis to reduce such large sets of individual gene names to identifiable biological functions (37).

We tested or network modules and submodules for enrichment in Gene Ontology (GO) (38) Biological processes. We used GO because it offers a comprehensive annotation of molecules over a wide range of processes thus serving as a valuable first approach. Overrepresentation was calculated with WEBGESTALT (39). Statistical significance threshold was set at *p* ≤ 0.05 after Benjamini & Hochberg FDR correction. For each module, we performed Over-Representation Analysis (ORA) based on FDR-corrected hypergeometric test over a category of genes whose functions are annotated in the Gene Ontology Consortium database GO (38) with the R package HTSanalyzeR (40), choosing a significance *p* ≤ 1 × 10^−5^.

## 3. Results

### 3.1. Inflammation-Related Network Has a Characteristic Expression Pattern for Each Module

**Figure 2:**
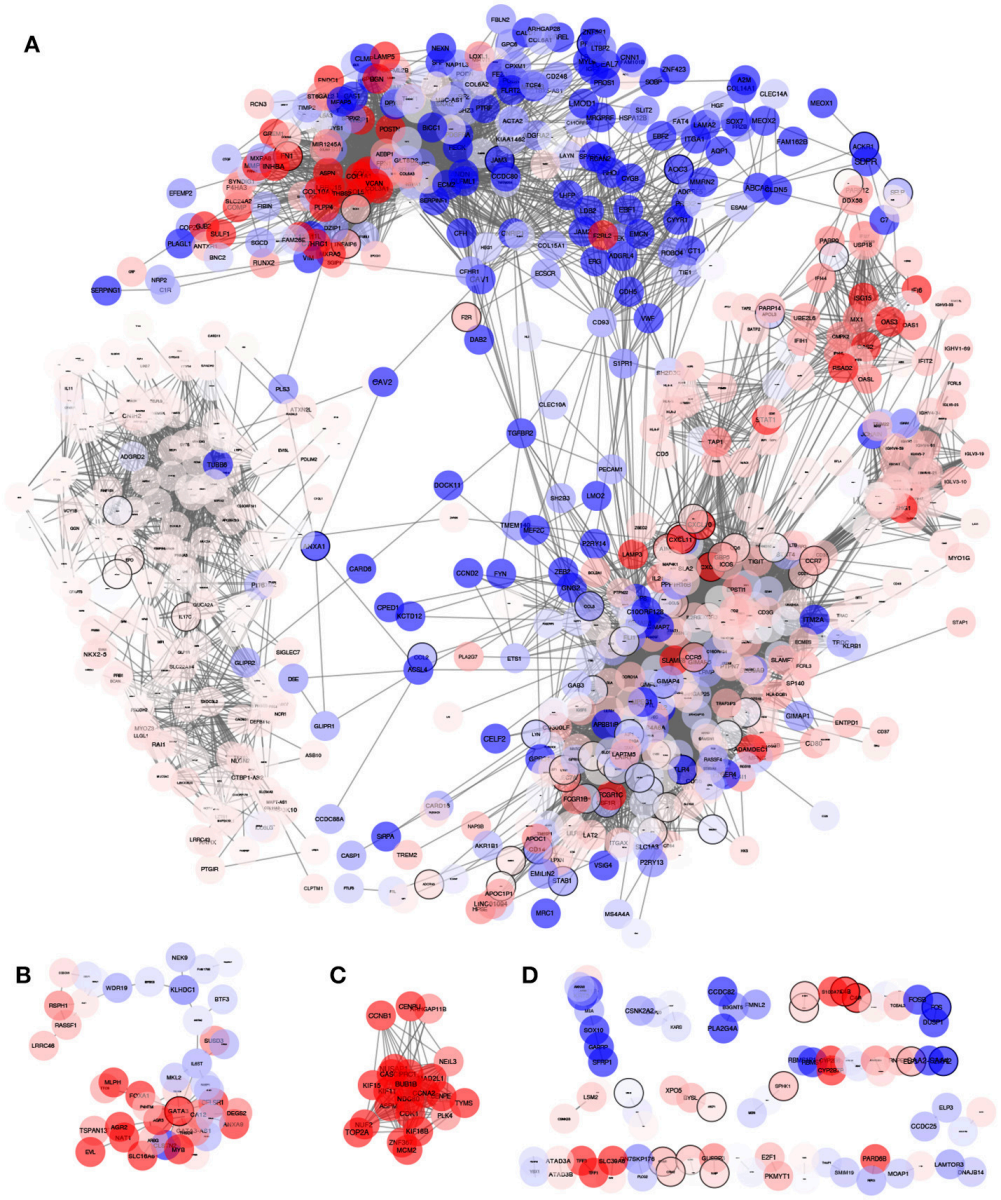
Network obtained from the MI highest interactions between inflammatory process genes and their first neighbors. The network consists of 942 genes and 10,000 interactions (edges). Node color represent the differential expression status compared to healthy mammary tissue. Red: Overexpressed, Blue: Underexpressed, White or pale color means no differential expression (−1≥*LogFch* ≤ 1). This network consists of many connected components of which three of them **(A–C)** consists of more than 10 genes. Indicated with **(D)** are small components of less than 10 genes. By observing the way nodes aggregate in the largest component **(A)**, it is evident that the network has an internal modular structure where differential expression levels seem to cluster with similar differential expression trends. We further explored how this network is organized and which known biological processes are being regulated.

The Inflammation-associated Gene Regulatory Network (IGRN) contains the top 10,000 interactions ordered by MI value; this IGRN has 942 genes with three connected components that contain more than 10 genes (Figures 2A–C and Supplementary File 1). Information about the DE status for each node was mapped to the network. This revealed a definite composition of DE genes for each component.

**Figure 3:**
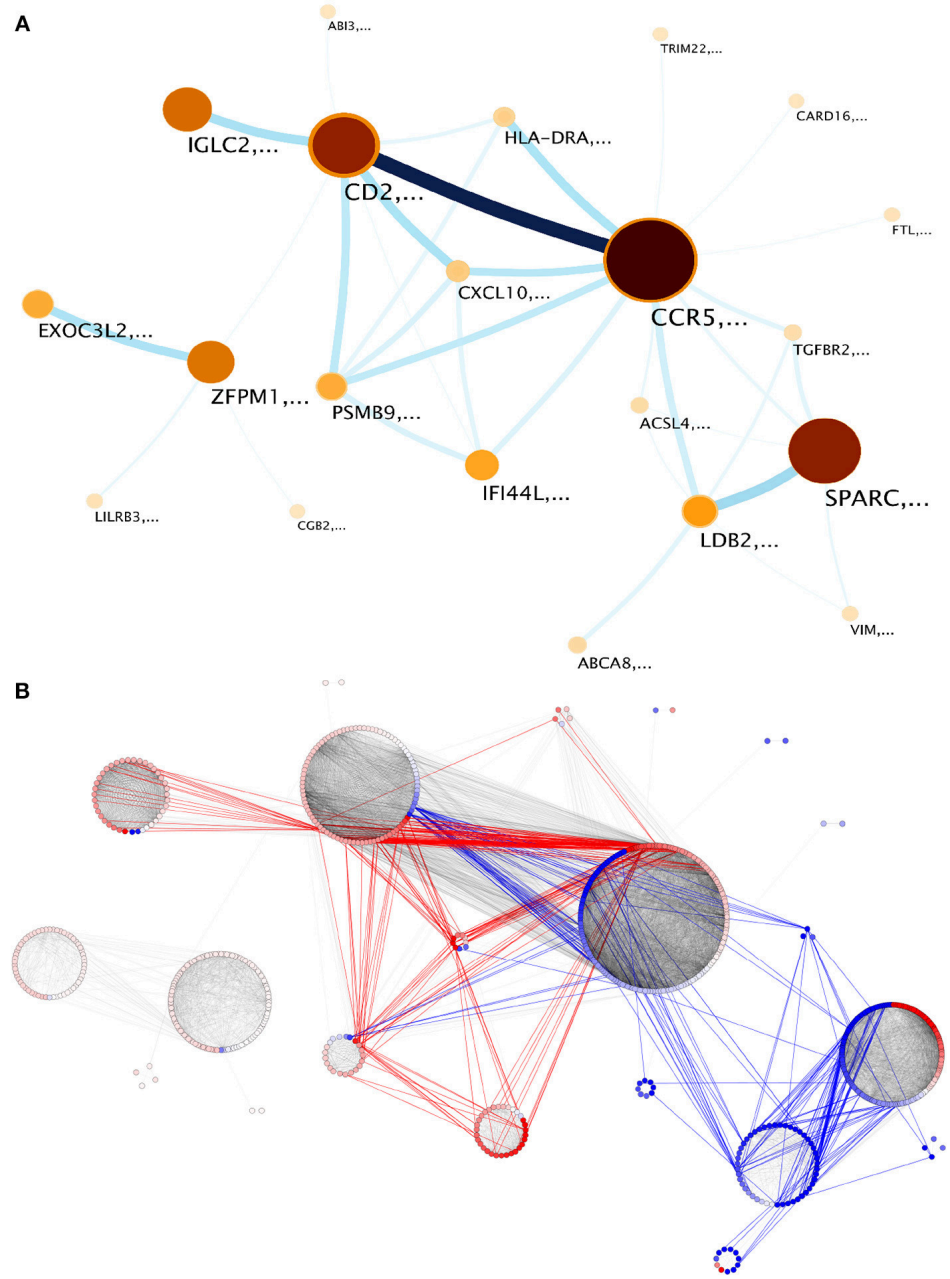
Modular structure of the largest component of the network. Twenty one submodules were identified. **(A)** infomap visualization. Nodes correspond to each detected module. Darkest colors and largest circles show those modules with more genes; link widths correspond to the flow between modules, proportional to the inter-module edges. Node labels refer to genes with highest page rank in the module. **(B)** inter-module edges preferentially link genes with similar expression patterns. Modules are visualized by differential expression. Red links are inter-module edges linking two overexpressed genes, blue links are analogous but regarding underexpressed genes, gray links show intra-module edges and inter-module edges between no-differentially expressed elements.

The largest component contains 787 out of the 942 genes in the network. We explored the module structure of this component, which revealed a hierarchical structure of 4 *first level* and 28 *second level modules* or *submodules* (Table 2 and Supplementary File 2). Modules and submodules were labeled based on the highest page-ranked gene (Figure 3A). Hereon, modules and submodules are indicated with a subscript after the name of its highest ranked gene i.e. *CCR*5_*m*_ and *CD*2_*sm*_.

**Table 2:** Modules and submodules in the largest component of the network.

**Module**	**Submodule**	**Number of genes**
***CCR*5_*m*_**		**376**
	*CCR*5_*sm*_	176
	*CD*2_*sm*_	87
	*IGCL*2_*sm*_	39
	*IFI*44*L*_*sm*_	31
	*PSMB*9_*sm*_	21
	*CXCL*10_*sm*_	9
	*HLA*−*DRA*_*sm*_	5
	*ABI*3_*sm*_	2
	*CARD*16_*sm*_	2
	*FTL*_*sm*_	2
	*TRIM*22_*sm*_	2
***SPARC*_*m*_**		**230**
	*SPARC*_*sm*_	152
	*LDB*2_*sm*_	41
	*ABCA*8_*sm*_	8
	*TMEM*204_*sm*_	8
	*SPARCL*1_*sm*_	7
	*CNRIP*1_*sm*_	6
	*VIM*_*sm*_	4
	*CARD*6_*sm*_	2
	*DZIP*1_*sm*_	2
***ZFPM*1_*m*_**		**173**
	*ZFPM*1_*sm*_	71
	*EXOC*3*L*2_*sm*_	58
	*JSRP*1_*sm*_	22
	*GUCA*2*A*_*sm*_	13
	*LILRB*3_*sm*_	4
	*ATXNL*2_*sm*_	2
	*CGB*2_*sm*_	2
	*ZNF*683_*sm*_	1
***ACSL*4_*m*_**		**8**

*Bold values in the number of genes column are the total gene number for the module and correspond to the sum of the gene numbers in al its submodules*.

When we observe the DE status in individual submodules, it becomes evident how it tends to display characteristic patterns for each submodule. For instance, submodules such as *LDB*2_*sm*_ are integrated by genes with a tendency to underexpression, and *IFI*44*L*_*sm*_ integrated by genes tending to overexpression (Figure 3B). In the case of *EXOC*3*L*2_*sm*_ and *ZFPM*1_*sm*_, all genes have a DE status of no change respect to normal mammary tissue. However, other submodules such as *SPARC*_*sm*_ and *CCR*5_*sm*_ show a mixed DE profile. Coordinated over and underexpression gene sets may account for modulation of opposing or conflicting pathways.

Now, based on the module detection method, a novel feature revealed is the information flow between the aforementioned modules, a measure of how much information is shared between them, which in turn is proportional to the number of inter-module edges, which is the number of links between genes belonging to different modules. The major contribution to this flow is by gene pairs with similar expression patterns: overexpressed genes join two different modules, and concomitantly underexpressed genes are responsible for the union between underexpressed modules. Interestingly enough, those modules with non-differentially expressed genes are connected between them (Figure 3).

The modular expression pattern of the IGRN suggests a coordinated activity of genes whose products (proteins) may be involved in the activation or inactivation of processes. It is not clear –although it may be possible– whether clusters of genes that are not differentially expressed could be related to processes of maintenance of mammary tissue, independent of the disease. The fact that genes included in the modules have a similar expression pattern concurs with the idea that network modules are not only a topological feature of the network, but also reflect a functional role in the definition of phenotypes (20, 23).

### 3.2. Network Modules Are Function-Specific

Modules are disjoint sets of nodes at the same hierarchical level. We performed overrepresentaion analysis for these gene sets, taking Gene Ontology:Biological Processes as the reference database. Statistically significant enrichment was found, but not for all modules and submodules.

**Figure 4:**
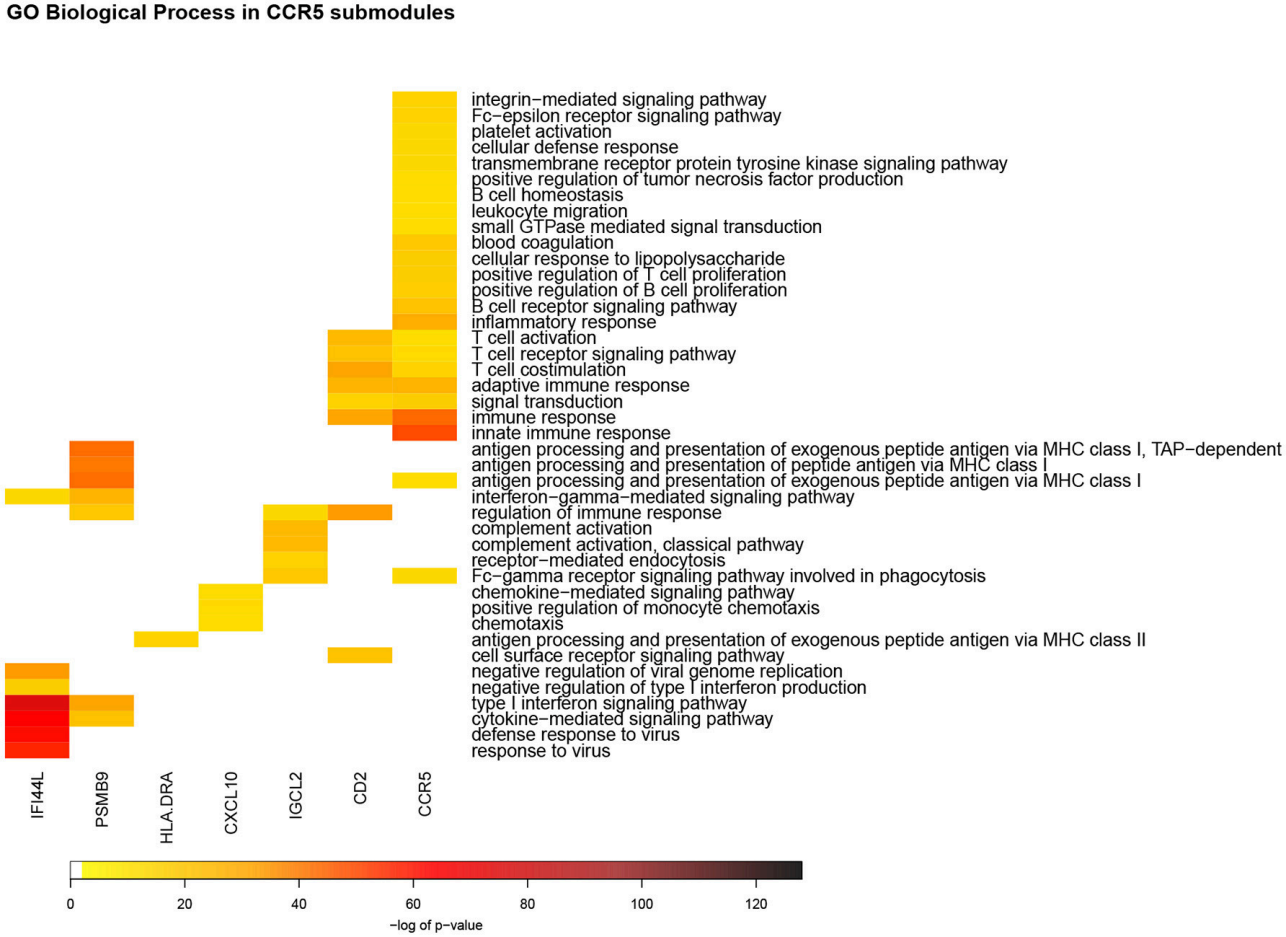
Heatmap of GO Biological process enrichments for *CCR*5_*m*_ submodules. In this representation, each column corresponds to a detected module according to the network structure. Each row is the enriched biological process in the aforementioned modules. The color code represents the enrichment *p*-value: red color takes account for the most significant values. In this picture can be observed that, despite *CCR*5_*sm*_, each module has a set of unique enriched processes, which could be related to the specificity of functions by each submodule.

Top enrichment scores for the *CCR*5_*m*_ include immune response, innate immune response, cytokine-mediated signaling pathway, adaptive immune response, also included is inflammatory response (Figure 4). *SPARC*_*m*_ top enrichments include extracellular matrix organization, cell adhesion, extracellular matrix disassembly, collagen catabolic process and angiogenesis. Since the inflammatory response process was enriched only in the *CCR*5_*m*_ we focused on the biological processes and pathways of its submodules.

Seven out of eleven *CCR*5_*m*_ submodules showed a statistically significant enrichment for GO Biological processes. Some of these enrichments were shared between submodules, with the largest category overlap between *CCR*5_*sm*_ and *CD*2_*sm*_ (Figure 4). These two submodules have numerous connections and are the two largest in terms of of gene and interaction counts (Figure 2).

### 3.3. CCR5 Submodule Is Responsible for Inflammatory Cell Recruitment

The largest submodule with 176 genes, is enriched for inflammatory response and other adaptive immune response processes. Genes coding for proteins involved in T lymphocyte signaling such as CD45, CD4 and CD28 which serve as coreceptors necessary for T cell activation. LYN, BTK, PIK3CD and VAV1 participate in the B cell signaling pathway, as well as in the Fcγ and Fcϵ signaling pathways through which receptor mediated endocytosis can be activated allowing antigen presenting cells to capture antigens bound to antibody molecules.

Said submodule contains genes for the receptors Fcγ RIIA and Fcϵ RIγ that participate in receptor mediated endocytosis signaling and recognize IgG and IgE class antibodies, respectively. Alternatively to the FcR pathways, phagocytic cells can produce molecules of the complement system that aid in the phagocytic process. C1q is a 18-mer integrated in a 1:1:1 proportion by the products of C1QA, C1QB and C1QC genes included in this submodule. Although components of the Complement System are produced in the liver, C1q is produced extrahepatically by macrophages and dendritic cells. Apart from its role in the classical complement pathway, C1q can bind to calreticulin exposed in membranes of apoptotic cells (41) and to antibodies that permeate and bind to intracellular antigens (42). This alternative functions give C1q an important role during apoptotic cell clearance by phagocytic cells (43) and even relates to autoimmunity development in certain contexts (44).

To induce effector function in CD4^+^ T cells it is necessary that antigens be presented by professional antigen presenting cells (APC) through MHC class II molecules. To achieve this, externally acquired antigens need to be processed in the phagosome and mounted in MHC-II molecules prior to exposure in the membrane of the APC. This submodule contains the gene HLA-DMB, responsible for the exchange of the clip peptide with antigen in MHC class II and shares numerous edges with the *HLA*−*DRA*_*sm*_. This submodule also contains NCF1, NCF2, NCF4, and CYBB, whose products are members of the NADPH oxidase complex isoform NOX2. NOX2 catalyzes the production of reactive oxygen species and proton consumption in phagosomes but is also responsible for a slower acidification of the endosomes, maintaining a higher pH which delays protein degradation by phagosomal proteases (45). Slow protein degradation in endosomes is associated with antigen cross-presentation by MHC class I molecules (46), which allows APCs to activate CD8^+^ T cells who normally attack infected or transformed cells through presentation of internally-only produced antigens.

Genes of the signal transduction process include chemokine receptors and chemokines involved in immune cell recruitment. CCR5 and its ligands CCL4 (also known as Macrophage inflammatory protein-1β), CCL5 (also known as RANTES), and XCL2, are invloved in T cell and monocyte recruitment. CXCR3 ligands CXCL9 (MIG), CXCL10 (IP-10), and CXCL11 (I-TAC) are part of the neighboring submodules *CD*2_*sm*_ and *CXCL*10_*sm*_. These cytokines are involved in leukocyte (CTLs, NKTs and macrophages) migration. CXCL10 and CXCL11 are also reported to be induced in response to interferon gamma (IFNγ) (47, 48) and are potent attractants for NK andactivated T Cells (49).

The molecules coordinated in this submodule suggest the coregulation of genes that elicit inflammatory cell recruitment mediated by cytokines, phagocytosis, antigen processing and presentation. These functions are at the beginning of adaptive immune response, and complemented by characteristic processes of effector immune cells, particularly cytotoxic CD8^+^ cells. This result will be discussed in the following section.

### 3.4. CD2 Submodule Contains Genes Characteristic of CD8+ T Cells

This submodule contains 87 genes, and shares most of its GO enriched processes with the *CCR*5_*sm*_. *CD*2_*sm*_ is highly connected to the *CCR*5_*sm*_ sharing 831 edges. Here are expressed cytokines CXCL9, a chemotactic cytokine for lymphocytes recognized by CXCR3 and CCL8 also known as monocyte chemoattractant protein 2 (MCP-2), recognized by CCR5 which serves as a chemoattractant to monocytes, which later can differentiate to phagocytic cells, and to attract lymphocytes, including T cells.

In this module we observe genes for the components of the T cell receptor, including the α, β and ζ chains as well as the CD3 γ, δ and ϵ chains, which amounts for the entire functional TCR assembly. Coexpressed genes for co-stimulatory molecules CD8A and ICOS are also found. *CD*2_*sm*_ contains IFNG, perforin (PRF1), and granzymes A, B, K, and H (GZMA, GZMB, GZMK, GZMH) genes and EOMES. IFNG is the only member of the interferon family that is found in the network and is a mediator in antiviral, antimicrobial and antitumoral responses, while perforin and granzymes are molecules responsible for the effector cytotoxic function of CD8^+^ and NK cells. EOMES codes for a transcription factor paralogue of T-bet that is induced in CD8^+^ T cells and has been associated to the expression of IFNG, perforin and granzime B in this cells (50).

For CD8^+^ T lymphocytes, antigens must be presented in MHC-I molecules. MHC-I presented antigens generally come from cytosolic degradation of proteins in the cytosol by the proteasome. Normal cells present endogenous peptides this way, but CD8^+^ T cells need to be activated by APCs that present externally acquired antigens processed in a distinct compartment called the phagosome. These external antigens can reach MHC-I molecules, a process known as cross-presentation (51). In an inflammatory environment, stimulation with interferon gamma induces the expression of alternative components of the proteasome modifying the way proteins are cleaved and thus altering the repertoire of presented peptides (52).

As we mentioned in the previous section, in this submodule we observe a funtional relationship with *CCR*5_*sm*_. This is backed by the fact that *CCR*5_*sm*_ triggers the adaptive immune response, meanwhile *CD*2_*sm*_ is related to effector functions, mainly involved in CD8^+^ cells response, which could imply a temporal relationship between both submodules, starting with *CCR*5_*sm*_.

### 3.5. PSMB9 Submodule Is Associated to MHC Class I Antigen Presentation

This submodule contains TAP1 and TAP2, along with PSMB8 and PSMB9 genes. These are functionally related to antigen processing and presentation via MHC class I. Antigens presented by MHC class I are mainly peptides that result from protein degradation in the cytosol by the proteasome. When cells are stimulated by cytokines like IFNG, genes of alternative components of the proteasome PSMB8, PSMB9 and PSMB10 are induced and its protein products replace components of the catalytic center of the proteasome to form what is known as the immunoproteasome (52).

The immunoproteasome has a distinct pattern of protein cleavage and the resulting peptides are more efficiently transported by TAP to the inside of the ER and more efficiently loaded in to MHC class I molecules. This allows the presentation of a distinct repertoire of antigens to CD8^+^ T lymphocytes and is important for the recognition and elimination of infected or transformed cells via the Cytotoxic T Lymphocyte (CTL) induced cell death in which perforin and granzyme play effector roles.

Previously (23), we observed in a basal breast cancer network a module formed by PSMB9, TAP1, and UBE26 genes among others. Said module was the only one with a significant enrichment in processes related to apoptosis. It is worth mentioning that in this case (undifferentiated breast cancer), we are able to observe a similar pattern with a network obtained from the unique connectivity pattern of inflammation-related genes and their neighbor genes. This result may suggest that the inflammatory process could be involved in the apoptotic response more closely than it has been reported (53).

### 3.6. HLA-DRA Submodule Is Related to MHC Class II Antigen Presentation

This submodule contains genes such as HLA-DRA, HLA-DRB, HLA-DQA, HLA-DQB, and HLA-DMA, which are known members of the MHC class II, and whose products are crucial in the presentation of antigens acquired in an extracellular fashion (Figures 3A, 4). MHC class II genes are expressed in professional APCs and are necessary to elicit antigen-specific responses mediated by CD4^+^ T cells. Genes in the MHC class II complex are induced in monocytes after IFNγ signaling that activates transcription factor CIITA (54).

### 3.7. IFI44L Submodule Is Related to Antiviral Responses and Is the Most Significantly Enriched

This submodule contains genes induced by interferon signaling. The protein products of many of these genes are known for their participation in antiviral responses. For example, OAS3 catalyzes the formation of 2′-5′ oligomers from ATP leading to the activation of RNAse 2 which degrades endogenous and viral RNAs. IFIH1 and IFIT1 are are cytoplasmic sensors of viral RNAs. PARP14 which is also coexpressed in this submodule is related to downregulation of IFNγ induced cytokines and enhances the transcription of STAT6-dependent genes (Figure 4).

### 3.8. IGLC2 Submodule Contains Genes for Antibody Production

This submodule consists of immunoglobulin chain genes, including heavy and light chains with variable and constant regions. Among the immunoglobulin isotype defining chains, this submodule contains the IGHA1, IGHG1, and IGHM heavy chains characteristic of IgA, IgG, and IgM antibodies. IGH1A is overexpressed, which is suggestive of IgG production, although it needs to be demonstrated at the level of protein presence in the tissue. In contrast to what is shown in *CD*2_*sm*_ where the T cell receptor components are coregulated, genes of the components of the B cell receptor are not present except for the IGHM chain (Figure 3).

### 3.9. CXCL10 Submodule Takes Part in Lymphocyte Recruitment

This submodule contains genes that code cytokines CXCL10 and CXCL11 which are CXCR3 ligands involved in lymphocyte migration as chemoattractants and are induced by IFNγ signaling (49). CCL2 and CCL8, known as MCP1 and MCP2 also are chemoattractants for leukocytes. The coordinated expression of these genes suggests an active inflammatory process where leukocytes are recruited to the tumor.

### 3.10. Association Between Inflammation and Adaptive Immunity

In order to quantitatively evaluate the association between inflammation and adaptive response, we performed a pathway deregulation analysis of our data by implementing the ‘Pathifier” algorithm (55) to identify sets of samples significantly deregulated (this is, samples with a pathway deregulation score, PDS > 0.4) in the inflammation and adaptive response related pathways.

From these analyses we found that out of our 641 tumor samples, a total of 395 (61.6%) were significantly deregulated in both, inflammation and adaptive response, whereas 210 (32.7%) were strongly deregulated in adaptive immunity but not in inflammation. Also 22 (3.4%) tumor samples resulted in deregulated inflammation without significant adaptive immunity changes and just 14 tumors (2.18%) were not significantly affected in adaptive immunity nor in inflammation.

It is noticeable that most tumors in our study are significantly affected in their adaptive immune responses (94.3%). This group includes most of the inflammation affected tumors (395 out of 417, or 94.7%). In contrast there are very few tumors without significant adaptive immune deregulation (36 out of 641 or 5.6% overall). Also scarce resulted tumors with notorious inflammation but with no significant adaptive immunity changes (22 out of 417 or 5.3%).

In order to further validate the aforementioned results, we have performed a similar analysis on an independent dataset including RNASeq data from the TCGA collaboration in breast cancer. The proportions are as follows. Out of 993 tumor samples, 973 samples (97.99% were strongly deregulated in both inflammation and adaptive response. Only 12 samples resulted significantly deregulated in inflammation with no noticeable deregulation in adaptive immunity (1.21%) and just 8 samples with affected adaptive immunity and no significant inflammation (0.81%). No sample among the 993 TCGA tumors was found without significant deregulation of both processes. Indeed, these figures support our claims of an important role of adaptive immunity in the breast cancer phenotypes (Supplementary Files 3, 4).

### 3.11. Key Genes in the Association Between Inflammation and Adaptive Immunity

Inflammation is a highly complex process, intrinsically including immunosuppressive feed-back signaling to activated T cells. For instance, expression of IL-10 and PD-L1 genes by activated myeloid cells is supposed to inhibit adaptive T cell response, whereas that of Il-12, for instance, should improve it. Similarly, adaptive immune response comprises a variety of immunosuppressive events, eventually elicited by T cell subsets expressing specific markers, e.g. FOXP3 for regulatory T cells. These events may be strongly associated to other relevant molecules in the inflammatory context, such as interferon gamma or Granzyme B.

To answer the previous questions, we have performed a generalized linear model (a form of logistic multivariate regression) for the association (membership) of the four groups of samples with the following results:

- FOXP3 expression is significantly associated (*p*-value = 0.0133, postive association) to adaptive immune response in breast tumors.
- FOXP3 and GZMB expression are significantly associated (*p*-value = 2.57 E-5 for FOXP3 and 8.14E-11 for GZMB, both postive associations) to ‘adaptive immune response plus inflammation” in breast tumors.
- The group with ‘no adaptive immune response nor inflammation” showed a discrete (although significant) negative association with FOXP3 expresion (*p*-value = 0.027).
- Interestingly, in all tumors FOXP3 expression is highly significant, positively associated to GZMB expression levels (*p*-value = 2.0E-16)

Adaptive immune response strongly depends on the B cell response. The association of the expression of genes related to B cell activation/Ig production and those involved in inflammation and T cell adaptive response is matter of interest. Being both adaptive immunity and inflammation extremely complex processes, comprising the concerted expression of hundreds of genes, we decided to evaluate that relationship by performing unsupervised learning (clustering) in the genesets associated to these processes in the four defined groups of tumor samples previously mentioned. The results are presented in the form of gene signatures displayed as clustered expression heatmaps (Supplementary File 5).

## 4. Discussion

### 4.1. Co-regulated Processes Suggest a Type I Inflammatory Microenvironment

**Figure 5:**
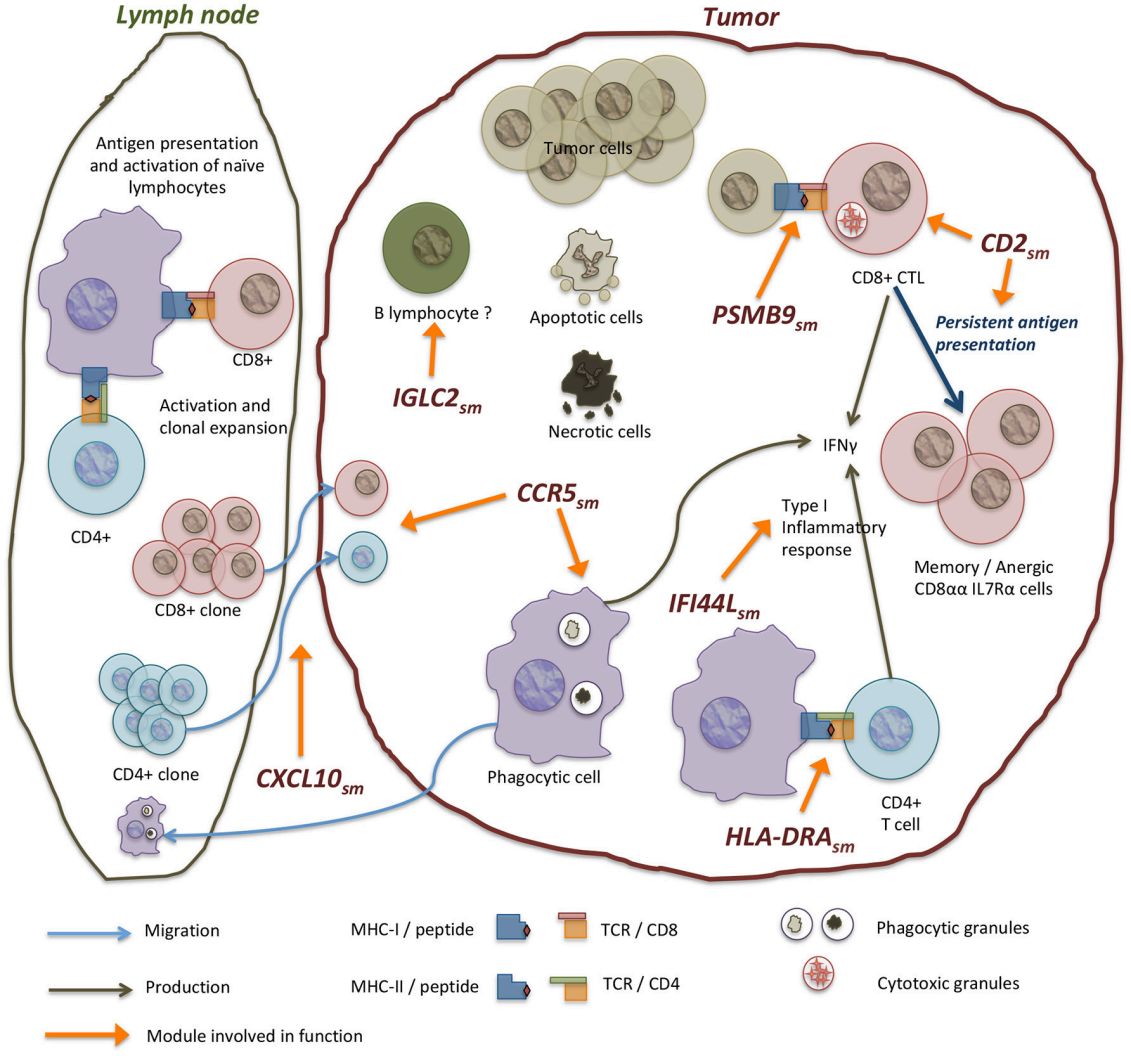
Modules in the network are enriched in processes of complementary biological functions. *CCR*5_*sm*_ contains genes involved in antigen acquisition via receptor mediated phagocytosis, endosome components, as well as a number of chemokines that mediate immune cell recruitment. *PSMB*9_*sm*_ contains genes of components of the immunoproteasome and MHC-I molecules involved in the presentation of internally produced antigens. The immunoproteasome is induced by IFNγ signaling and is associated with a response to intracellular infections and transformation in tumor cells. *HLA*−*DRA*_*sm*_ contains MHC-II genes involved in antigen presentation to CD4^+^ T lymphocytes. Other modules like *IFI*44*L*_*sm*_ also contain interferon-induced genes annotated with antiviral functions. *CD*2_*sm*_ has genes involved in T lymphocyte function, including T cell receptor components and co-receptor molecule CD8A as well as other genes related to T cell cytotoxic function like perforin and granzymes. Inside tumors, antitumor mechanisms coexist with tolerance mechanisms which impair tumor immune destruction. Our results suggest an active process of tolerance where cytotoxic CD8^+^ lymphocytes turn to anergic/memory cells that no longer fight the tumor. This is supported by the coregulation of CD8A and IL7R genes in *CD*2_*sm*_ but not of CD8B. Other modules like *IGLC*2_*sm*_ contain genes of immunoglobulin chains, including constant and variable chains that may be indicative of the presence of B lymphocyte infiltrates.

An inflammatory reaction is elicited in response of alterations in the tissue, recognized in the form of danger signals. An initial production of signaling molecules is followed by the recruitment of immune cells which in turn integrate signals and produce new molecules to adjust the response. This is indeed what our results suggest (see Figure 5). Our findings are also consistent with the presence of a type I immune response in breast cancer. Type I responses are characterized by the production of IFNγ and the recruitment of cytotoxic T lymphocytes, which includes CD8^+^ T lymphocytes (56). This type of response is associated to virus or intracellular parasites, as well as transformed cancerous cells and culminates in the elimination of targeted cells.

Our dataset included samples from patients with developing tumors that received no neoadjuvant treatment. In this context, where type I inflammatory response is associated to good prognosis and tumor elimination, it seems paradoxical that a *favorable immune response* has been related to tumor growth.

### 4.2. Inflammation and the Shaping of the Microenvironment

The cellular composition of breast cancer tissue includes cancerous cells as well as immune cell infiltrates. Among the latter, our subnetwork suggests the presence of phagocytic and antigen-presenting cells (i. e. by the presence of MHC class II in *HLA*−*DRA*_*sm*_ and Fc receptors in *CCR*5_*sm*_) and T CD8 and CD4 cells (TCR components CD3D, CD3E, CD3G, TRAC, TRB-C1, TRB-C2, and CD8α correceptor in *CD*2_*sm*_, as well as CD4 and CD28 correceptors in *CXCR*5_*sm*_). These are indicative of antigen-specific immune responses.

Naïve T lymphocytes that have escaped deletion by central tolerance mechanisms do not readily migrate to tissues. Instead, they first circulate and home to lymph nodes where they are exposed to antigens previously captured and presented by professional antigen presenting cells (57). If an antigen is recognized by the TCR the cell goes through changes in gene expression, proliferates and becomes an activated lymphocyte (58) (Figure 5). This process is called priming. Primed cells are able to enter inflammed tissues and exert their effector functions when they encounter their specific antigen. In some circumstances effector functions can be minimized or suppressed in the presence of antigen, what is known as peripheral tolerance.

Tolerance to self-antigens is necessary to avoid autoimmune tissue destruction, while tolerance to antigens derived from food and microbiome components keeps inflammation at bay. However, in the context of infections and cancer, tolerance may be part of the contributing factors of the pathology of chronic disease.

The effector function of CD8^+^ T cells is to kill cells infected with intracellular parasites (virus or bacteria such as mycoplasma) or transformed cells. This is achieved by means of membrane disruption and activation of the apoptosis extrinsic pathway. The latter is done by directed vesicle secretion of perforin and granzymes (PRF1, GZMA, GZMB, GZMH, GZMK in *CD*2_*sm*_) over the target cell. In order to achieve this, an activated CD8^+^ cell must recognize its antigen presented in MHC class I molecules expressed in the surface of the target cell (59). Given that CD8^+^ cells respond to antigens produced an processed inside presenting cells, priming of naïve CD8^+^ by APCs must be made by cross presentation of externally acquired antigens (57).

In a CD8^+^ antitumoral reponse, antigens can be acquired in the tumor by phagocytic cells, then transported to secondary lymphoid organs and cross presented to prime CD8^+^ T cells after which activated CD8^+^ cells migrate to inflammation sites and ideally kill cells that present the antigen. The activation of CD8^+^ cells results in a pattern of clonal expansion, followed by clonal reduction and survival of memory cells, all this in consonance with the apperance, presentation and eventual clearance of the antigen. However, if antigen is persistently presented and it is recognized with high affinity, clonal reduction and effector function are altered (60). Chronic exposure to antigen is associated to downregulation of TCR and CD8 signaling components, and the expression of anergy-specific genes (i.e. Itch, Cbl-b and GRAIL) (60).

Redmod and Sherman (60) proposed a model that explains how chronic antigen exposure can lead to persistence of anergic T CD8^+^ cells. In this model, strong signaling through the TCR eliminates many of the activated cells either throug IL7Rα receptor downregulation or a decrease in antiapoptotic molecules. Meanwhile, strong TCR signaling in the surviving cells produces an increase in free calcium levels triggering anergy and a reduction in the Ras-ERK proapoptotic signaling.

In our network CD8A and IL7R genes are coregulated in the *CD*2_*sm*_. IL7Rα and CD8αα are associated to the survival and development of memory CD8^+^ T cells (61, 62). This is particularly intriguing, given that our expression matrix contains both CD8A and CD8B genes for the CD8α and CD8β subunits of the CD8 correceptor, but only CD8A is included in the network. This may point out to purely CD8αα signaling, which is hypothesized to exert a weaker TCR signaling response. Anergic CD8 T cells need to be exposed to antigen to maintain anergy, and when antigen is removed, cells recover its response capacity.

For the construction of this network, interactions and genes were obtained from a background that included the whole genome (gene set defined by the HGU133 plus2 platform) and the strength and ranking of the statistical dependencies between gene pairs was not known a priori. Only 76 out of a total of 942 genes in the network are annotated as part of the inflammatory response process, which represents 8% of the network gene count. Meanwhile 694 genes in the network are annotated as part of the immune response process, that is 74% of the genes in the network.

The whole network was constructed taking into account all inflammation-associated genes, the modular and submodular structure of this network groups specific functional processes for particular modules (Figure 4). This could reflect the compartmentalization of immune response, activating sets of genes depending on the type of response that should be triggered. In these terms, general modules could be active as a first response under cellular damage or an external influence. For example, CCR5 and SPARC modules are associated to two different general cell processes: immunity (CCR5) and extracellular remodeling (SPARC). Our interpretation here is that modules partially reflect cell types present in the tumor microenvironment and that are sampled as part of the biopsy. This is specially suggested in the *CD*2_*sm*_ where a number of genes characteristic of CD8^+^ T lymphocytes are grouped together.

Immune response is a keystone in cancer behavior. The fact that several immune-related processes are divided into coexpressed compartments could optimize the immune response depending on the specific type of stimulus. A clear example of the aforementioned is the distinction between MHC class I and II in two separated and barely communicated submodules: *PSMB*9_*sm*_ is associated to MHC class I and *HLA*−*DRA*_*sm*_ takes account for MHC class II. Along with this, *IFI*44*L*_*sm*_ is associated to viral response (Figure 4). This effect has been previously observed by our group (23, 63, 64). Again, the topology of modules in the network is strongly associated to specific and separated functions.

Concomitantly, the interconnection between submodules inside the modules could be associated to fluxes of information between processes or cooperativity between modules. This is the case of *CCR*5_*sm*_ and *CD*2_*sm*_. In Figures 3A,B it is possible to observe that the strongest link between submodules occurs in this pair. Furthermore, the number of common enriched processes between modules is the highest. Processes related to T-cell activation and adaptive immune response are shared between these two submodules, indicating possible cooperation mechanisms under the need to stimulate T-cell activity.

Interestingly enough, ZFPM1 module, despite the fact that it contains 173 genes does not result statistically enriched for any biological process at our chosen significance threshold. This results remarkable since the belonging genes are not differentially expressed (white genes clustered in left side of Figure 2). These genes may not be part of the global response that cell performs under an external stimulus.

The results obtained from our network of inflammation-associated genes suggest that in primary, untreated breast cancer tumors, the balance between antitumor response and immune tolerance involving CD8^+^ T cells is tipped in favor of the tumor. A possible mechanism being the induction of tolerance and anergization of these cells by persistent antigen exposure. This hypothesis is supported by the presence of modules in the network with genes that enrich processes and pathways related to antigen acquisition, processing and presentation, as well as genes characteristic of CD8^+^ T effector cells.

## 5. Summary

In breast cancer, several kind of efforts must be achieved in order to understand the complex interplay between factors shaping cancerous phenotypes. In this sense, the modular analysis of the network structure observed here, provides us with an alternative framework of the aforementioned interplay between specific modules that take account of particular biological processes that are activated in response to external stimuli. Furthermore, the observed results highlight the compartmentalized structure of co-regulated genes that may act and behave together in a well-defined time and space.

The global genetic regulatory program observed in the form of a gene network may provide insights to understand the complex mechanisms behind relevant processes for cancer development such as inflammation and the adaptive immune response. To disentangle the association between these two events by using an information theoretical approach have allowed us to analyze at an extremely detailed level such regulatory programs. Said efforts will surely increase our knowledge on the complexity of this dismal disease and provide important tools for a personalized medicine (65) aimed to produce better diagnostic, prognostic and therapeutics for breast cancer.

## Author Contributions

TV-C implemented methods, performed calculations, analyzed results, drafted the manuscript. SA-C performed calculations, and contributed to the analysis of results. JE-E contributed to the analysis of results, collaborated in the discussion, reviewed the manuscript. EH-L conceived the project, contributed to the analysis of results, collaborated in the discussion, reviewed the manuscript. All authors read and approved the final version of the manuscript.

### Conflict of Interest Statement

The authors declare that the research was conducted in the absence of any commercial or financial relationships that could be construed as a potential conflict of interest.

## Supplementary Material

Supplementary File S1 A text file containing the edge list or connectivity map for the gene regulatory network (a Cytoscape SIF file).

Supplementary File S2 A text file with the definition of the modules and submodules for the largest component of the network (an Infomap MAP file).

Supplementary File S3 A multi-page PDF file with heatmaps presenting gene signatures related to the expression of genes related to specific pathways in the adaptive immunity and inflammation groups of samples in our original dataset.

Supplementary File S4 A multi-page PDF file with heatmaps presenting gene signatures related to the expression of genes related to specific pathways in the adaptive immunity and inflammation groups of samples in the validation (TCGA) dataset.

Supplementary File S5 A multi-page PDF containing gene expression boxplots for relevant genes in the different groups of inflammatory response.
