# Supplementary material for: Unveiling the Link Between Inflammation and Adaptive Immunity in Breast Cancer
Source: Front Immunol. 2019 Jan 29;10:56. doi: 10.3389/fimmu.2019.00056 (PMC6362261; doi:10.3389/fimmu.2019.00056)
Supplement: Supplementary File S3 — A multi-page PDF file with heatmaps presenting gene signatures related to the expression of genes related to specific pathways in the adaptive immunity and inflammation groups of samples in our original dataset. [file Data_Sheet_3.pdf]

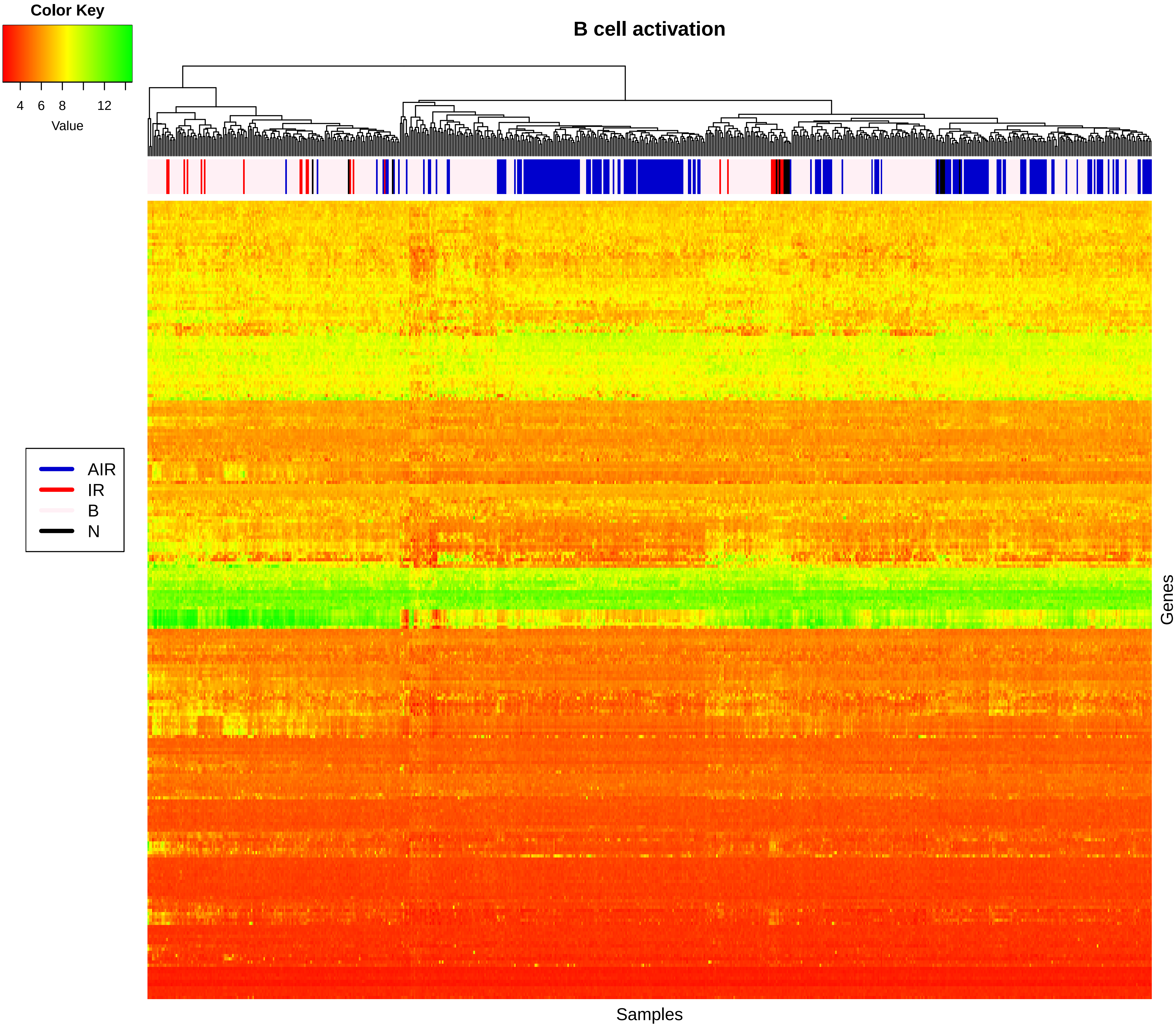
Color Key
4
6
8
12
Value
B cell activation
AIR
IR
B
N
Genes
Samples

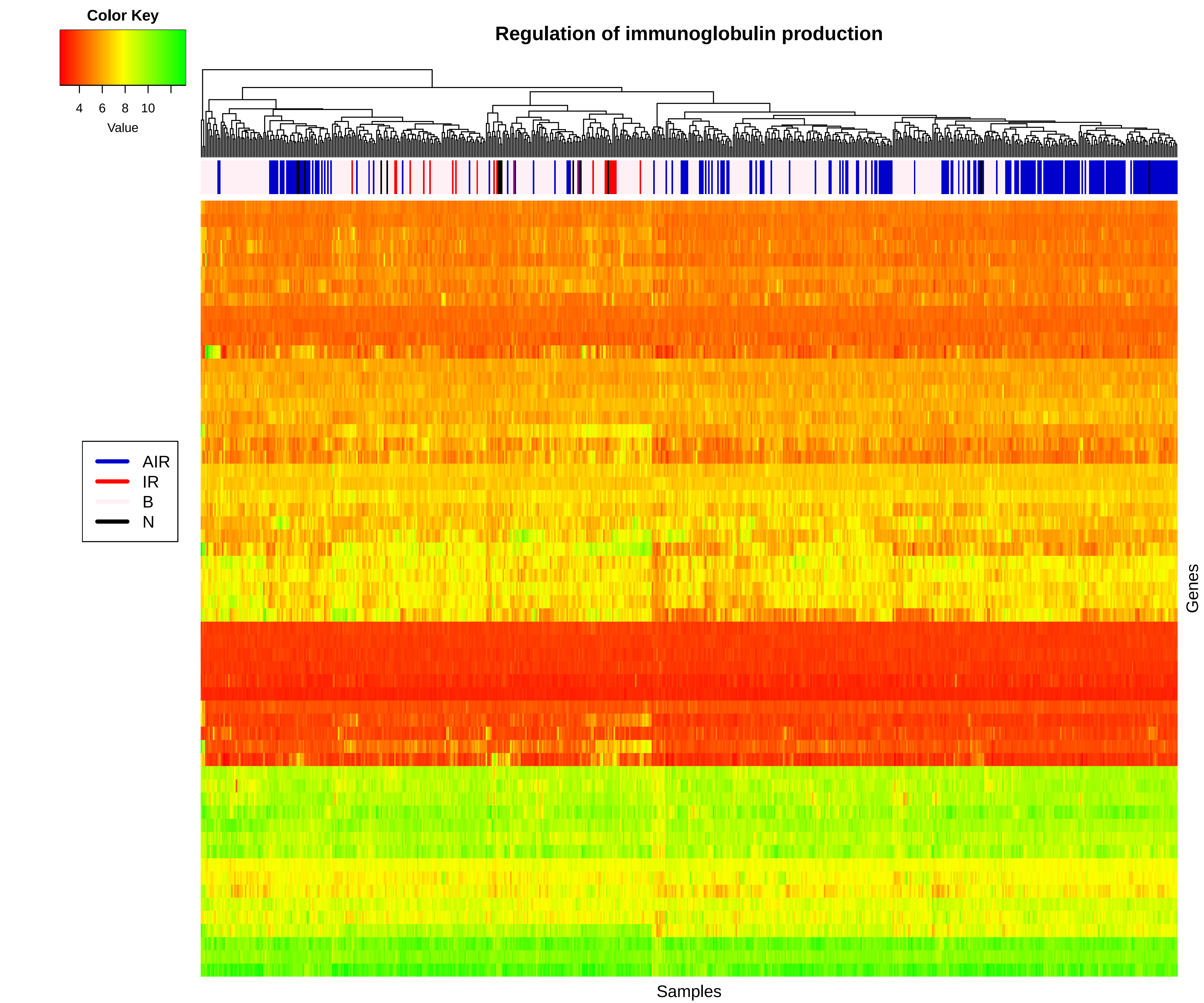
Color Key
4
6
8
10
Value
Regulation of immunoglobulin production
AIR
IR
B
N
Genes
Samples

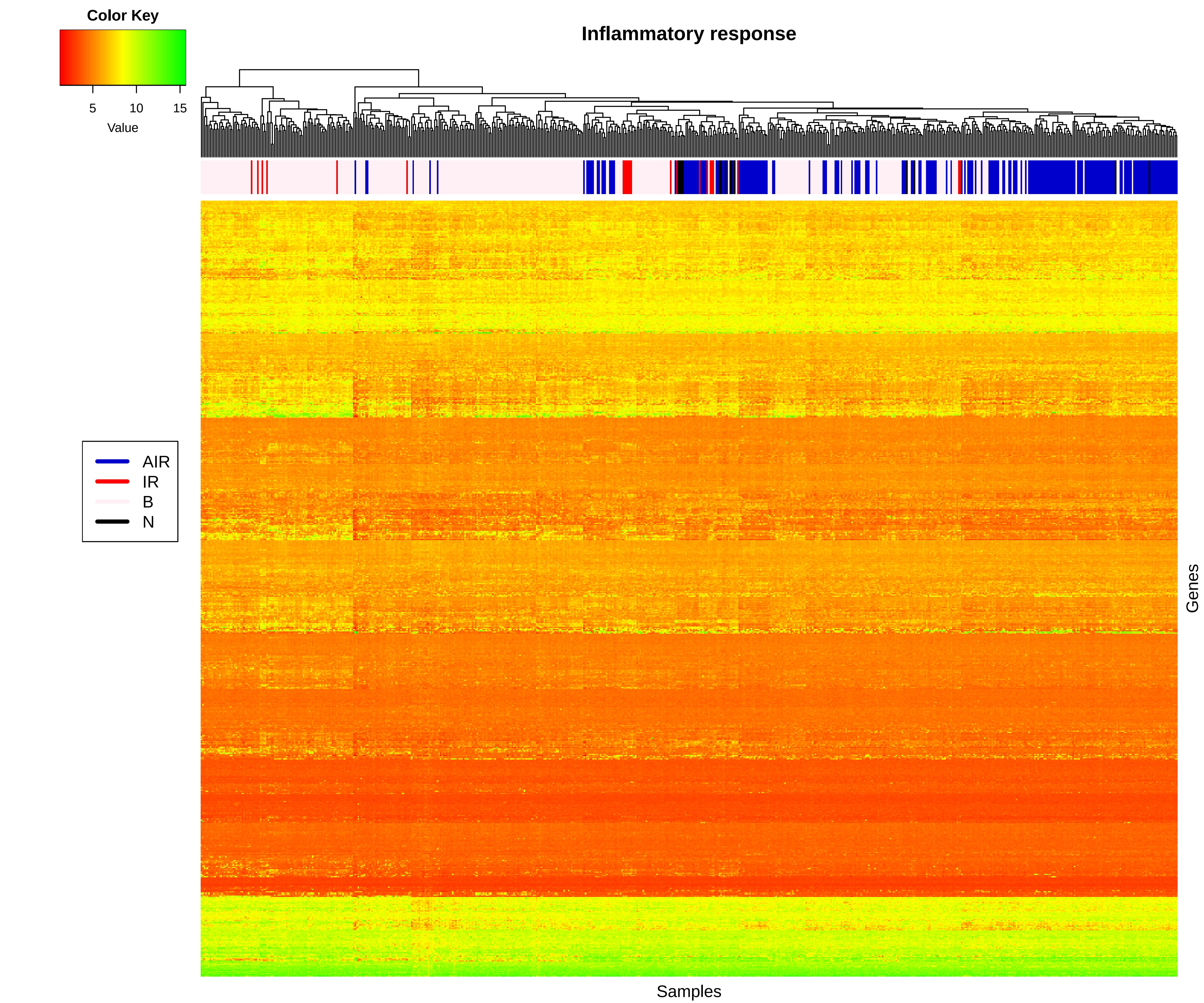
Color Key
5
10
15
Value
Inflammatory response
AIR
IR
B
N
Genes
Samples

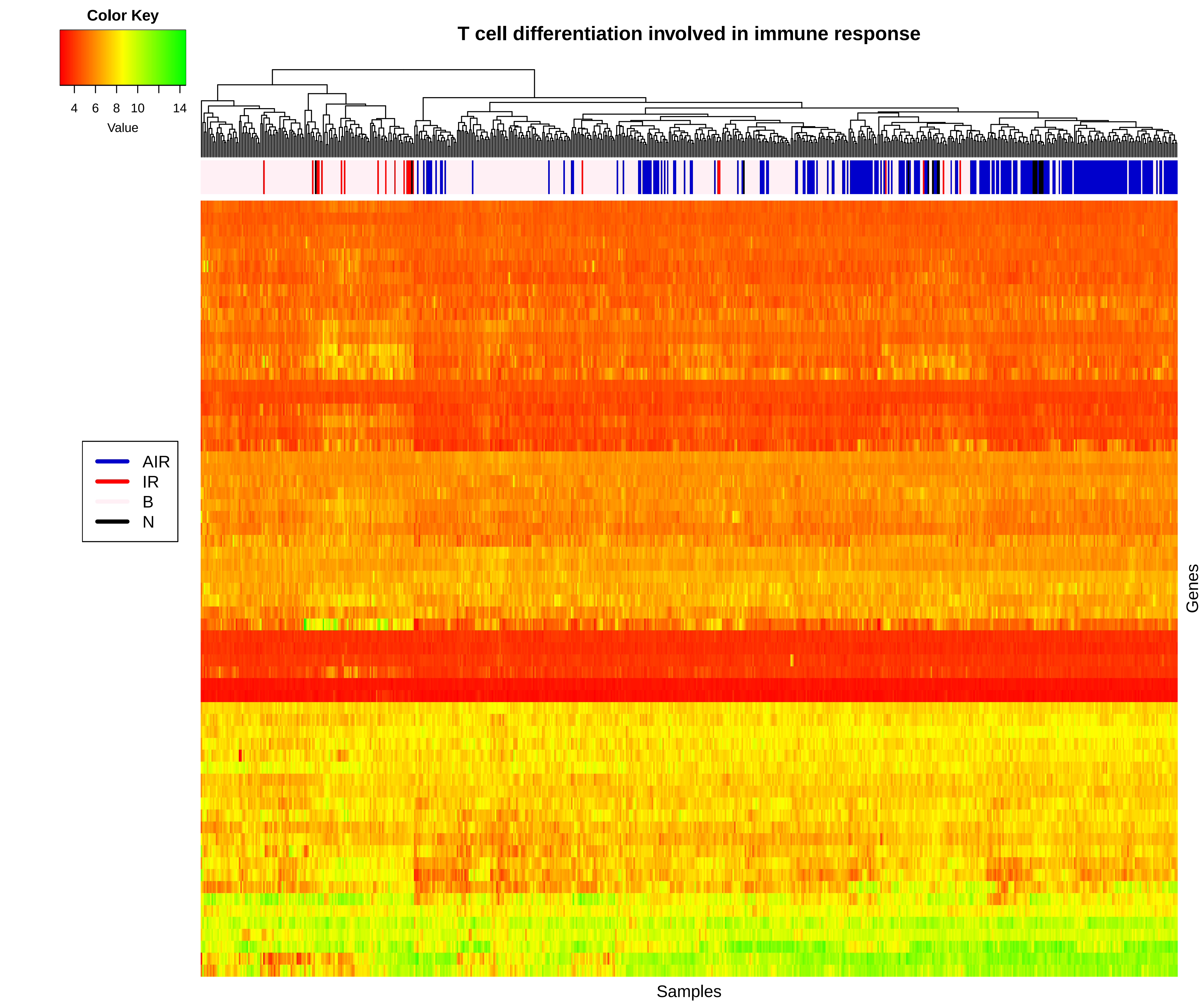
Color Key
4
6
8
10
14
Value
T cell differentiation involved in immune response
AIR
IR
B
N
Genes
Samples
