# Supplementary material for: Unveiling the Link Between Inflammation and Adaptive Immunity in Breast Cancer
Source: Front Immunol. 2019 Jan 29;10:56. doi: 10.3389/fimmu.2019.00056 (PMC6362261; doi:10.3389/fimmu.2019.00056)
Supplement: Supplementary File S4 — A multi-page PDF file with heatmaps presenting gene signatures related to the expression of genes related to specific pathways in the adaptive immunity and inflammation groups of samples in the validation (TCGA) dataset. [file Data_Sheet_4.pdf]

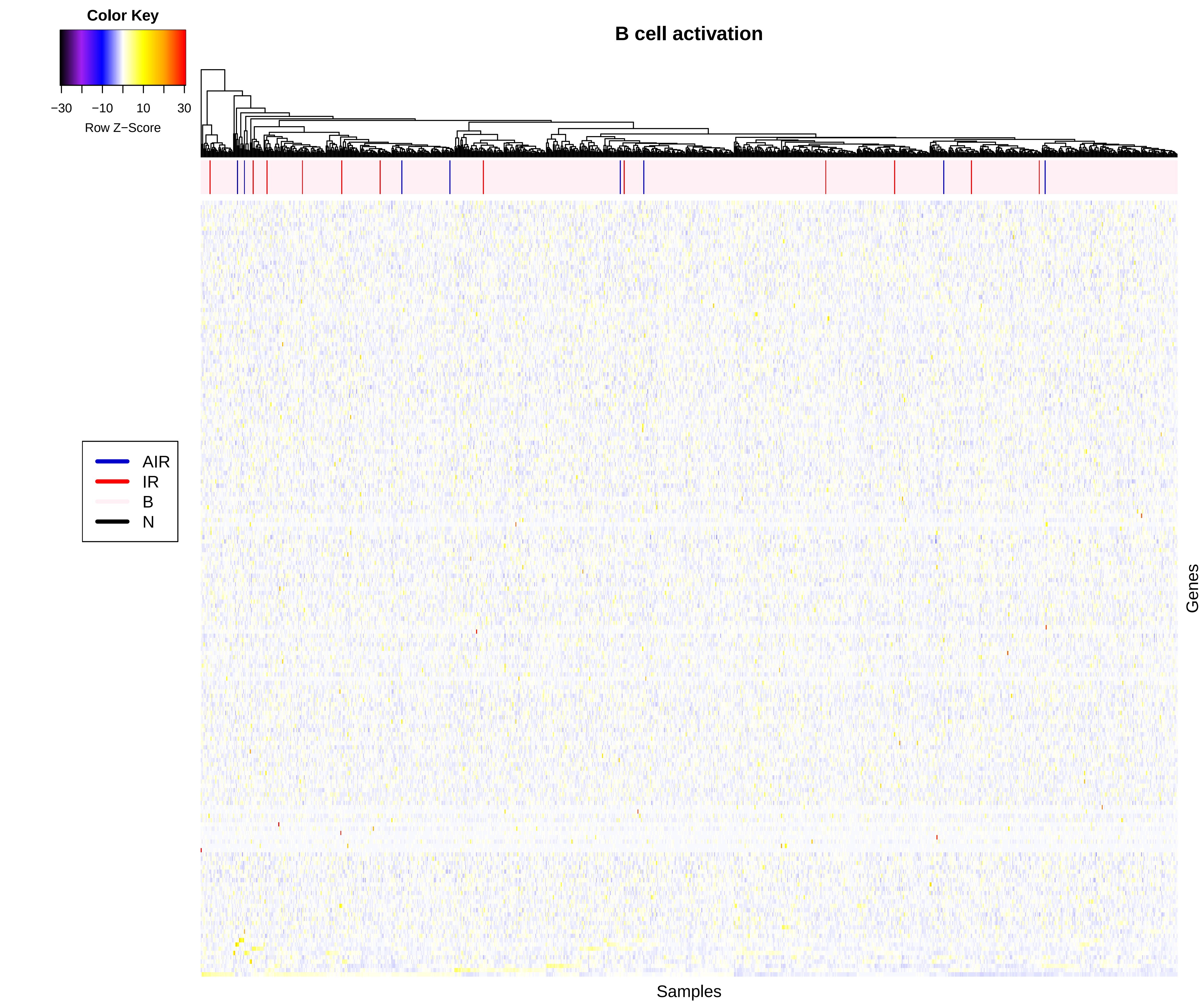

Color Key
−30
−10
10
30
Row Z−Score
B cell activation
AIR
IR
B
N
Genes
Samples

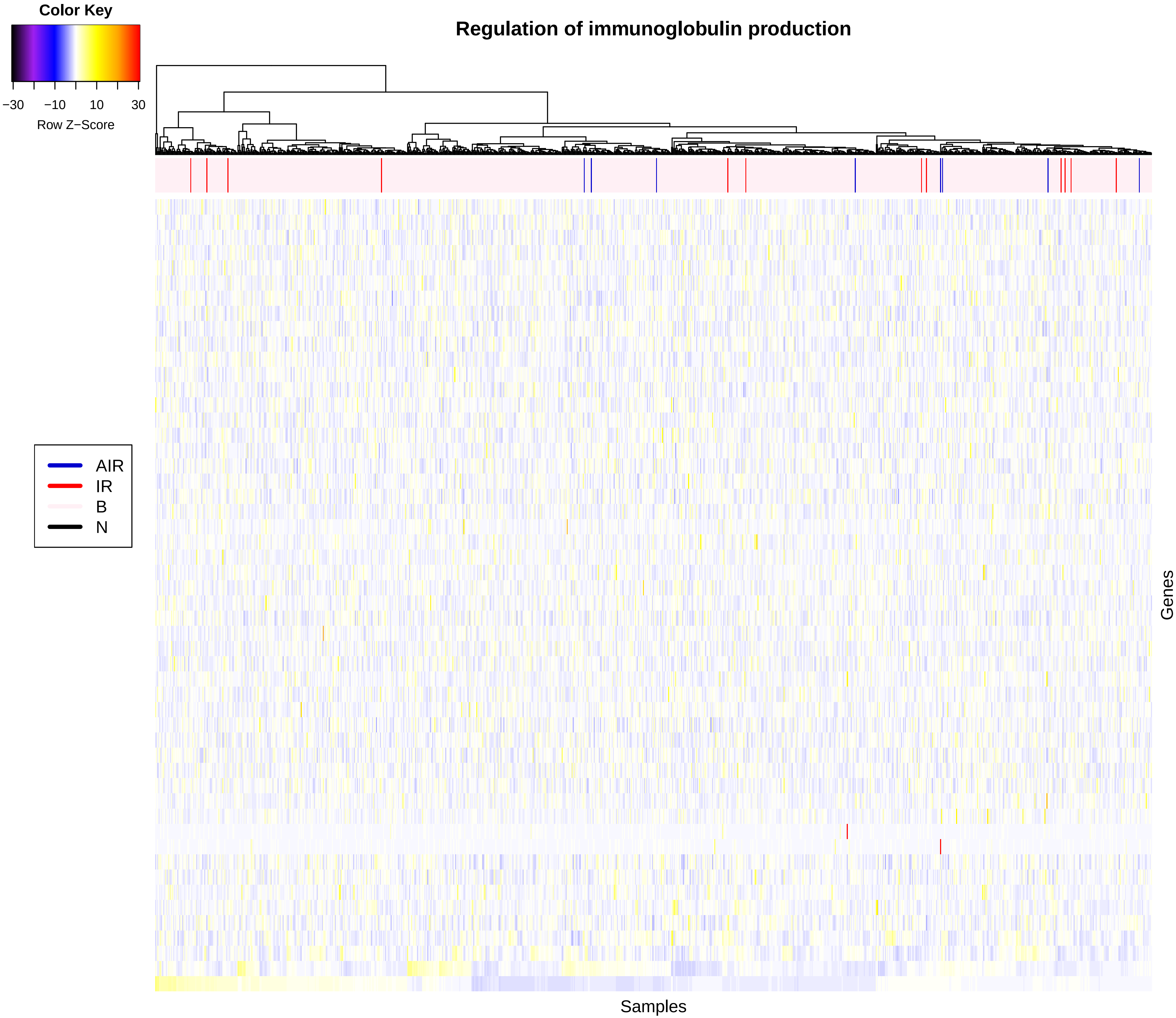

Color Key
−30
−10
10
30
Row Z−Score
Regulation of immunoglobulin production
AIR
IR
B
N
Genes
Samples

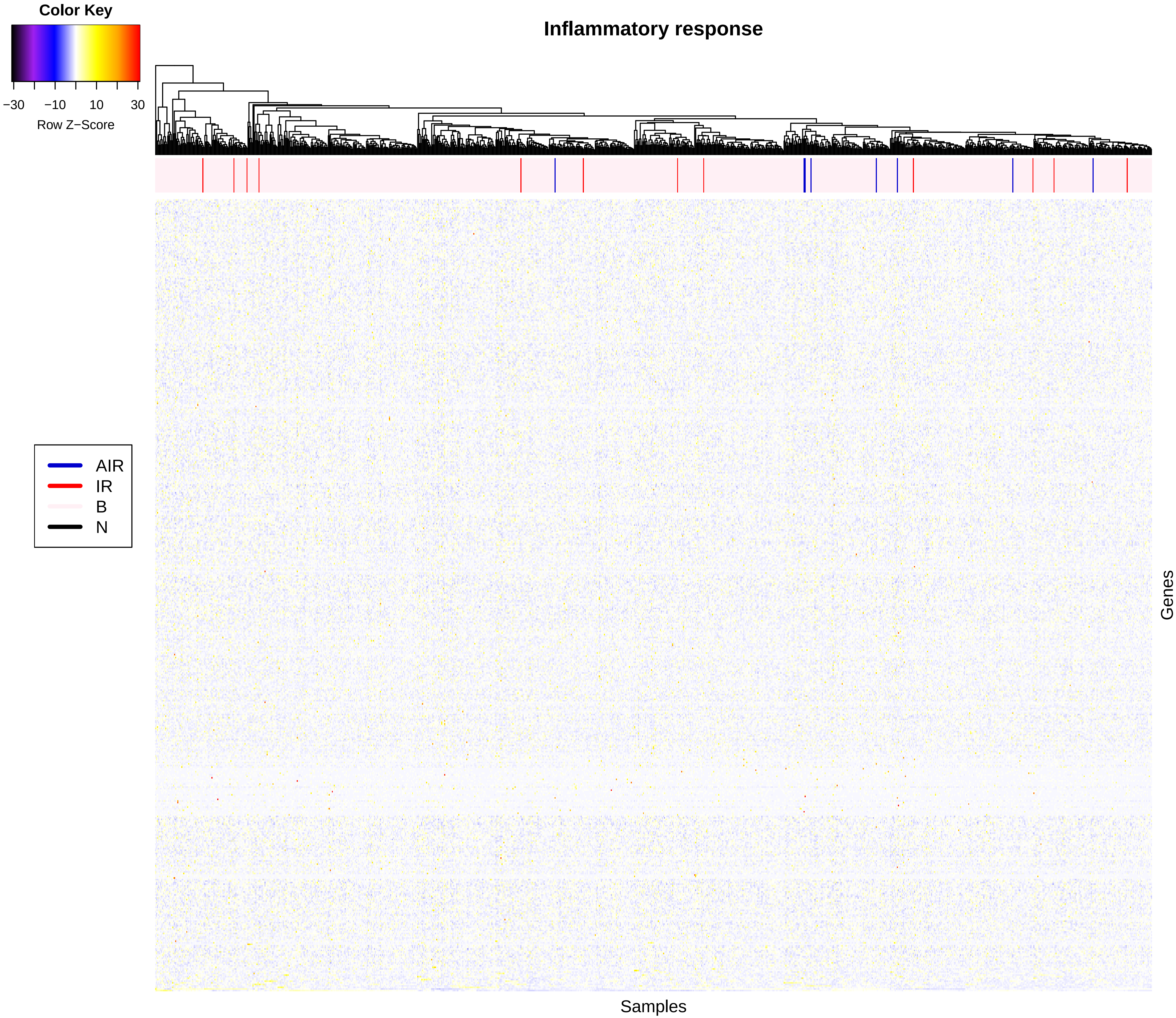

Color Key
−30
−10
10
30
Row Z−Score
Inflammatory response
AIR
IR
B
N
Genes
Samples

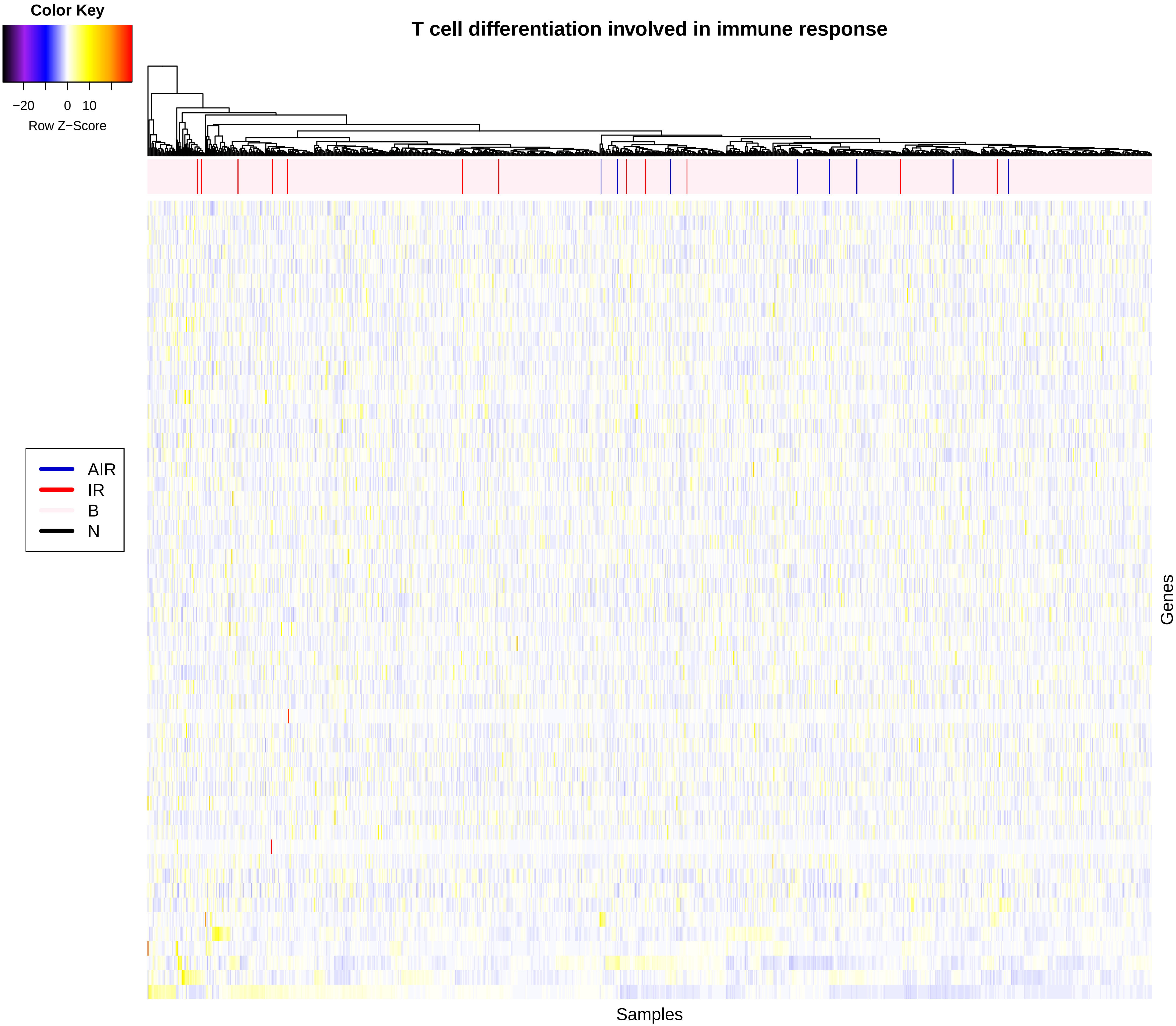
Color Key
−20
0
10
Row Z−Score
T cell differentiation involved in immune response
AIR
IR
B
N
Genes
Samples
