# Supplementary material for: Unveiling the Link Between Inflammation and Adaptive Immunity in Breast Cancer
Source: Front Immunol. 2019 Jan 29;10:56. doi: 10.3389/fimmu.2019.00056 (PMC6362261; doi:10.3389/fimmu.2019.00056)
Supplement: Supplementary File S5 — A multi-page PDF containing gene expression boxplots for relevant genes in the different groups of inflammatory response. [file Data_Sheet_5.pdf]

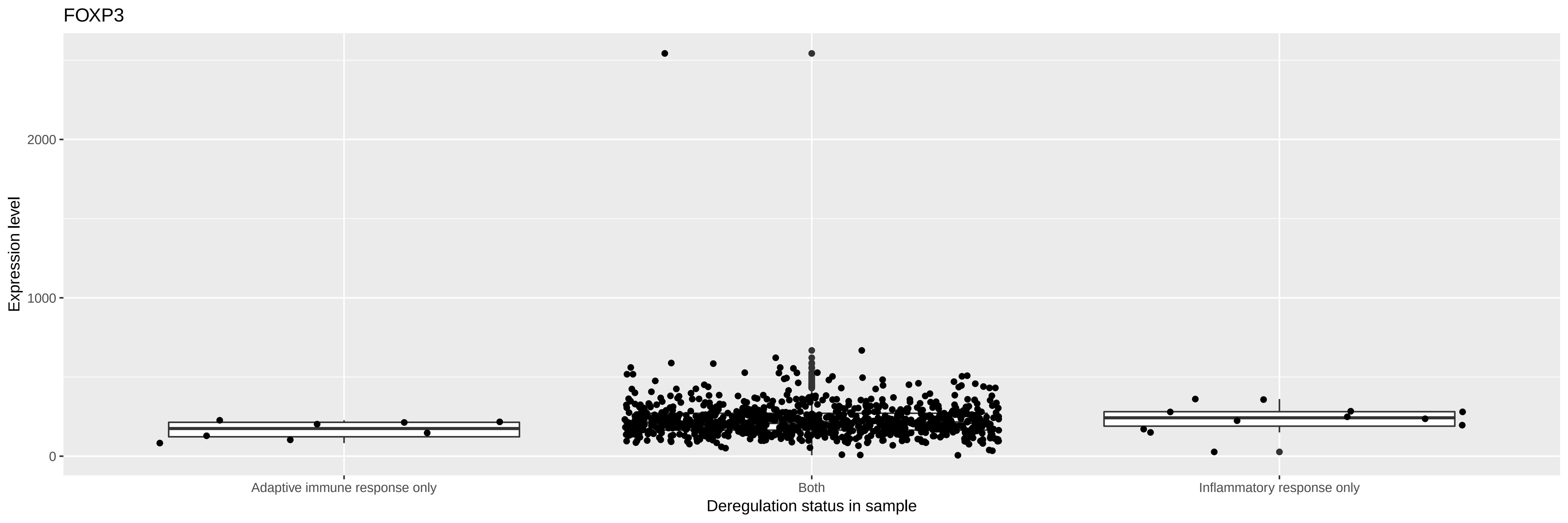

FOXP3
Expression level
2000
1000
0
Adaptive immune response only
Both
Inflammatory response only
Deregulation status in sample

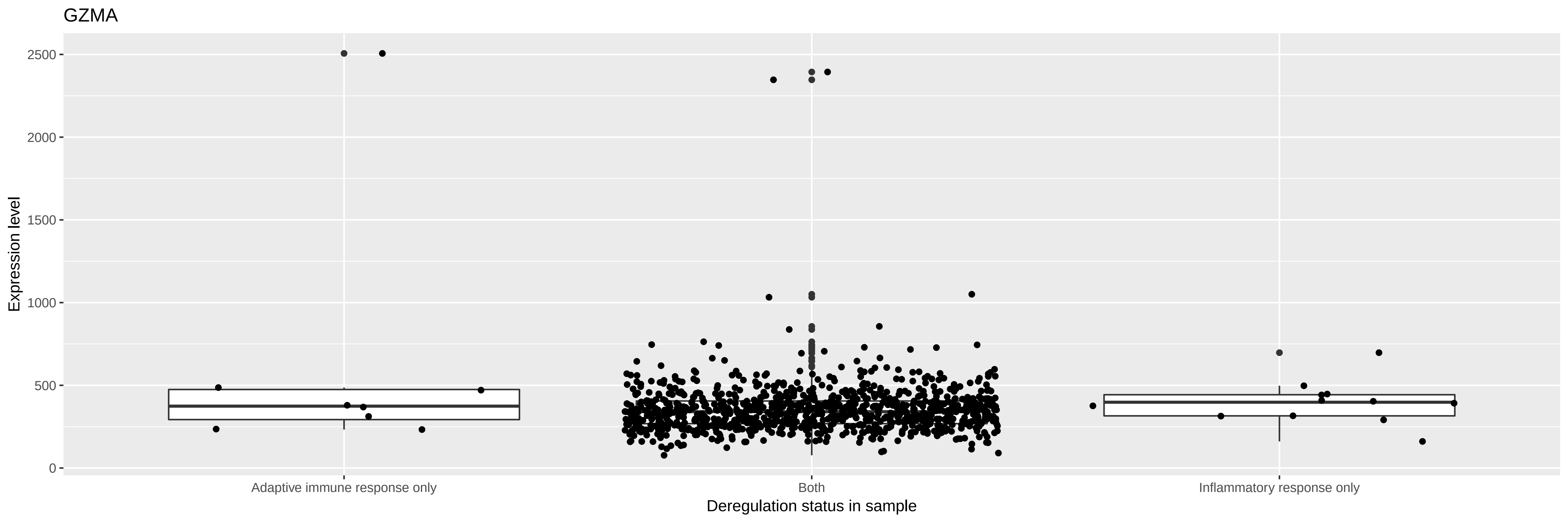
GZMA
Expression level
2500
2000
1500
1000
500
0
Adaptive immune response only
Both
Inflammatory response only
Deregulation status in sample

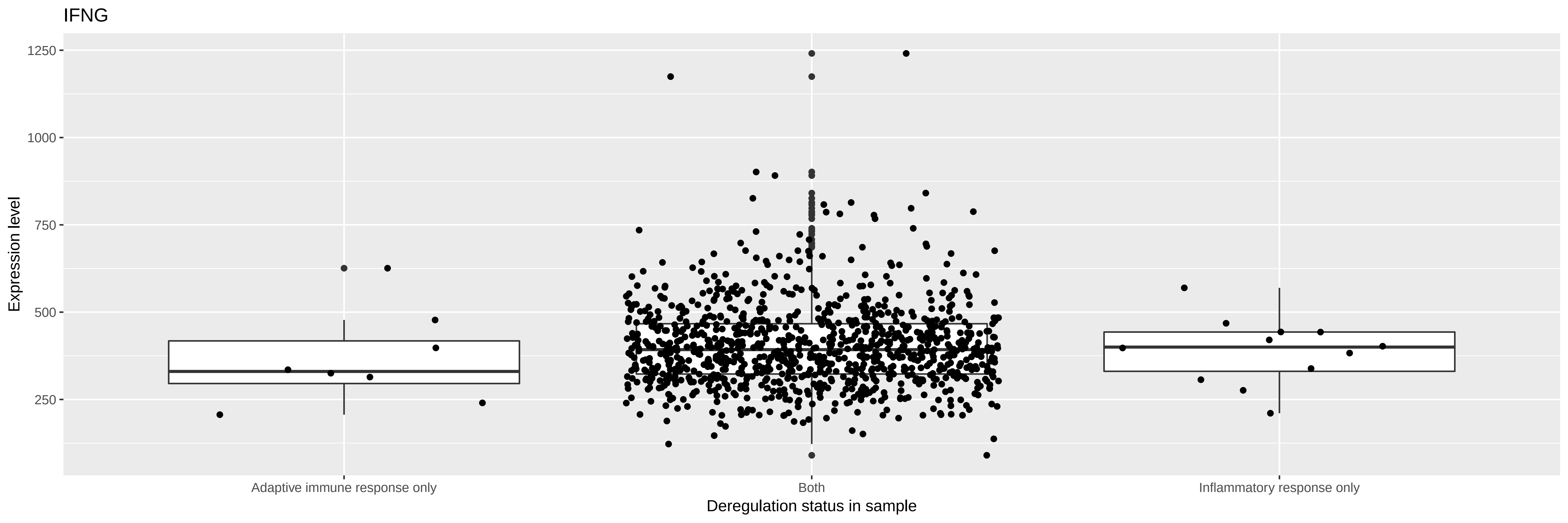
IFNG
Expression level
1250
1000
750
500
250
Adaptive immune response only
Both
Inflammatory response only
Deregulation status in sample

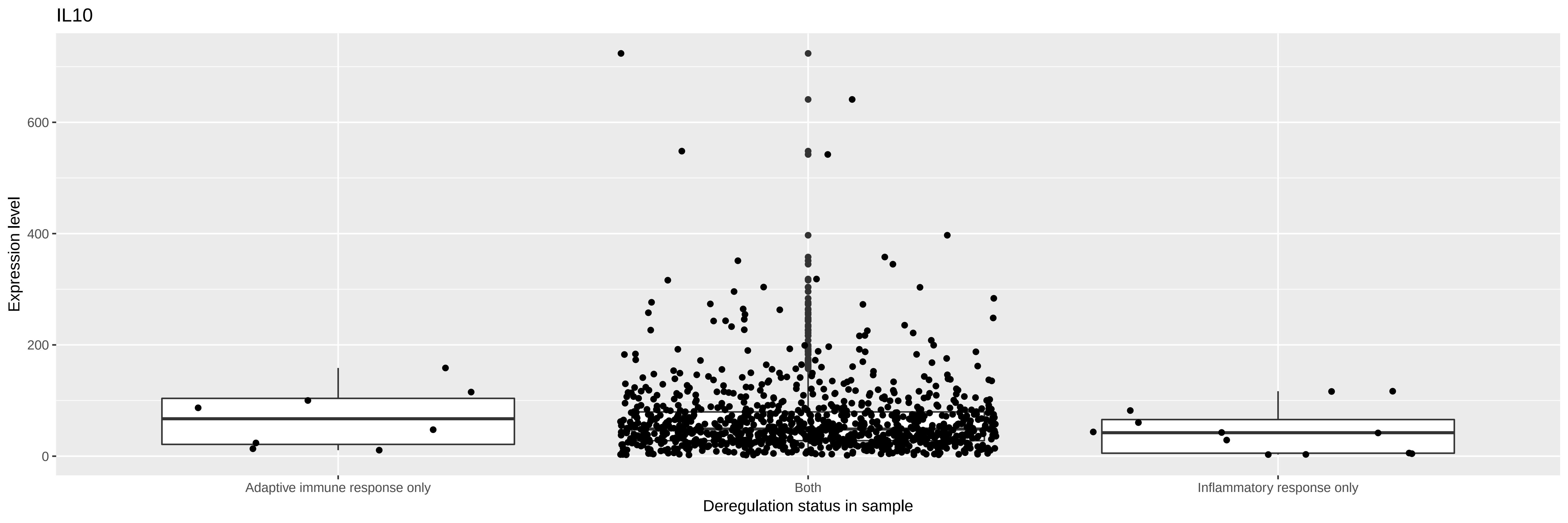
IL10
Expression level
0
200
400
600
Adaptive immune response only
Both
Inflammatory response only
Deregulation status in sample

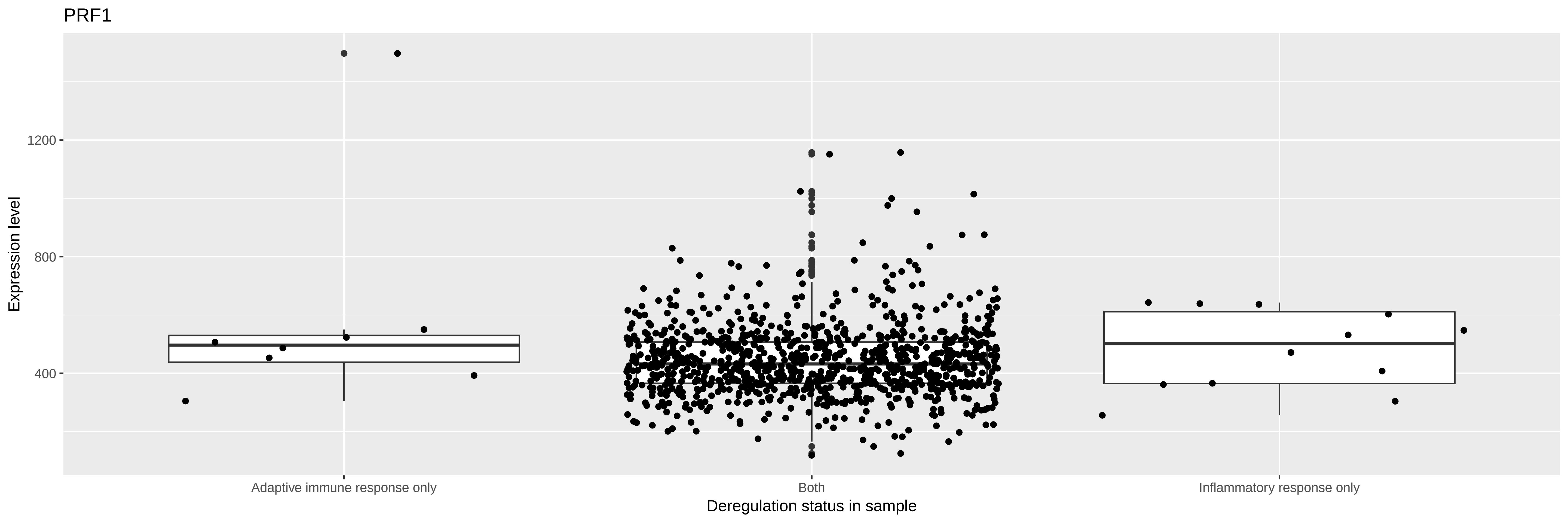
PRF1
Expression level
1200
800
400
Adaptive immune response only
Both
Inflammatory response only
Deregulation status in sample
